# An assessment of self-rated life satisfaction and its correlates with physical, mental and social health status among older adults in India

**DOI:** 10.1038/s41598-023-36041-3

**Published:** 2023-06-05

**Authors:** Mahadev Bramhankar, Sampurna Kundu, Mohit Pandey, Nand Lal Mishra, Adarsh Adarsh

**Affiliations:** 1 Department of Bio-Statistics and Demography, International Institute for Population Sciences, Mumbai, India; 2 Centre of Social Medicine and Community Health, Jawaharlal Nehru University, New Delhi, India; 3 Department of Survey Research and Data Analytics, International Institute for Population Sciences, Mumbai, India; 4 Laboratory of Population Health, Max Planck Institute for Demographic Research, Rostock, Germany

**Keywords:** Diseases, Geriatrics, Public health, Quality of life

## Abstract

Life satisfaction refers to the assessment of one’s own life in terms of self-perceived favourable qualities. It is an integral part of healthy and successful course of ageing. It is widely associated with the health status and social well-being. The present study attempted to determine the constructing factors of self-rated life satisfaction, such as socio-demographic, physical, social, and mental well-being of older adults. We analysed information from the Longitudinal Ageing Study in India (LASI-1), the initial phase conducted during 2017–18 for the population of older adults in India. We applied descriptive statistics for prevalence assessment and association was checked using chi-square test. Further, to determine the adjusted outcome of predictor covariates on the likelihood of an individual being satisfied from life estimated by applying hierarchical multiple logistic regression models. Several noteworthy affirmations on the relationship between the socio-demographic variables and health risk behaviours with life satisfaction were observed. The results were consistent with studies showing change in life satisfaction subject to the state of physical and mental health, presence of chronic diseases, friends and family relations, dependency, and events of trauma or abuse. While comparing respondents, we found varying degrees of life satisfaction by gender, education, marital status, expenditure and other socio-economic features. We also found that besides physical and mental health, social support and well-being play a pivotal role in achieving higher life satisfaction among older adults. Overall, this work contributes to the study of the subjective well-being of older adults in India based on self-reported levels of life satisfaction and further narrows the gap in knowledge about associated behaviour. Hence, with on-going ageing scenario, there is need for multi-sectorial policy-oriented approaches at individual, family, and community level, which helps to take care of older-adults’ physical, social, and mental health for the betterment of healthy ageing.

## Introduction

Life satisfaction is an integral part of healthy ageing and is an emerging public health concern. The ageing process is irreversible and degenerative, which comes with changes in the health of older adults, lifestyle behaviour, socio-demographic background, social support and mental health. The world is facing major challenges due to the increase in the ageing population since the 65 years old population outnumbered 700 million globally in 2019 and is expected to double to 1.5 billion by 2050^1^. This increasingly older population demands better health and satisfying life to be happy. While ageing is a universal phenomenon, it varies across subgroups. The sense of fulfilment and happiness could come at old age for some, while others could lament their physical disabilities or decline in their overall well-being^2,3^.

Veenhoven^4^ defined ‘life satisfaction’ or ‘happiness’ as “*the degree to which an individual judges the overall quality of his or her life as a whole favourably*”^4^. Older persons who have suffered from bad health mostly tend to have low life satisfaction. It is majorly associated with the person’s health status, be it physical or mental^5^. Active ageing, which optimises overall health and enhances the quality of life^6^, is a major part of life satisfaction. Regular participation in social activities, cultural spirit, physical activities and civic affairs is part and parcel of active ageing. With increasing age, older adults tend to become less active in social participation. Studies suggest that individuals who were not involved in any social activity were probably inactive and had a lower quality of life than socially active individuals. Simultaneously, continuous participation in social activities is associated with good health and better life satisfaction^7^.

Several characteristics, such as social, economic status, marital status, education level, social support^8–10^, cognitive and mental health, affects life satisfaction^11,12^. The changing patterns of society and demographics in India have led to the disintegration of families. There have been shifts from joint to nuclear families, profoundly affecting aged people^13^. Very limited studies have shown that people living with spouses and children are more satisfied with life than people living alone^14–17^. Life satisfaction is also related to health predictors, including self-reported health and health behaviour^18^. According to WHO, four factors influence life satisfaction levels directly- physical health, mental health, social support, and environment^19^. Functional abilities like activities of daily living (ADLs) and instrumental activities of daily living (IADLs) were also identified as contributing factors toward life satisfaction^20,21^.

Life satisfaction and the factors contributing to well-being in old age constitute a major concern for the older population and gerontological research. Older adults who enjoy positive social relationships do not have any chronic illness and have good living arrangements. The level of life satisfaction of older adults can be improved by providing them with a safe, healthy and trustworthy environment and proper health facilities. In case they are suffering from some chronic disease and arrangements of better living arrangements should be made so that they cannot feel like a burden on society but as a productive resource of society. Thus, the present study examines the status of life satisfaction according to constructing factors, such as socio-demographic, economic, health behaviour, physical health, social health and support, and mental health among older adults in India.

## Methods

Data for this study is collected from the first wave of the Longitudinal Ageing Study in India (LASI) 2017–18. It is a full-scale national survey of scientific investigation of the health, economic, and social determinants and consequences of population ageing in India^22^. The first wave of the survey covered 72,250 older adults aged 45 and above and their spouses, irrespective of their ages, even under 45 years, across all states and union territories of India except Sikkim. A multistage probability stratified area cluster sampling design was adopted to arrive at the eventual units of observation. Detailed information on the survey design, instruments used, and data collection can be accessed from LASI India Report^22^. This study focused on eligible respondents aged 45 years and above only. The total sample size for this study was 65,562 older individuals of age 45 years and above.

### Outcome variables

The outcome variable for this study, i.e. self-rated overall life satisfaction, is based on the response of older adults age 45 and above to the question, “Please think about your life as a whole. How satisfied are you with it? Are you completely satisfied, very satisfied, somewhat satisfied, not very satisfied, or not at all satisfied?”. The dependent variable is coded in two different ways as per its utility. In the case of cross-tabulation, we have used three categories recoded as “high life-satisfaction” for ‘completely satisfied’ and ‘very satisfied’; “medium life-satisfaction” for ‘somewhat satisfied’ and “low life-satisfaction” for ‘not very satisfied’ and ‘or not at all satisfied’. However, dichotomised version of this variable is used for multiple logistic regression analysis where ‘completely satisfied’, ‘very satisfied’ and ‘somewhat satisfied’, were recoded as “satisfied” while ‘not very satisfied’ and ‘not at all satisfied’ were recoded as “unsatisfied”.

### Explanatory variables

Explanatory variables included in this analysis can be categorised under five heads– background characteristics, health risk behaviours, physical health indicators, mental health, and social well-being. Background characteristics include age (45–59, 60–74 and 75 + years), sex (male and female), place of residence (rural and urban), marital status (currently married, widowed, divorced, separated, deserted or others), religion (Hindu, Muslim, Christian and others), caste/tribe (scheduled tribes, scheduled caste, other backward classes and others), an education level (respondents with- no schooling, less than 5 years of schooling, 5 to 9 years of education and 10 or more years of schooling), work status (currently working, worked in the past but currently not working and never worked), monthly per capita expenditure MPCE (poorest, poorer, middle, richer and richest quintiles) and geographical regions (north, south, east, west, north-east and central regions).

Health risk behaviours include smoking tobacco (ever smoked or used smokeless tobacco coded as yes and no), drinking alcohol (ever drinks alcoholic beverages coded as yes and no) and being physically active (coded as inactive and active). Physical activity was measured through a set of questions under 2 heads- moderate and vigorous physical activities. Moderate physical activity includes the drawing water from a well, engagement of respondents in cleaning the house, washing clothes, fetching water, gardening, bicycling at a regular pace, and floor or stretching exercises walking at a moderate pace. While for vigorous activity, they were asked about their involvement in running or jogging, cycling, swimming, going to a health centre/gym, farm work, heavy lifting, fast bicycling, cycling with loads, digging with a spade or shovel, and chopping. As per WHO norms, those who were either engaged in at least 150 min throughout the week (moderate physical activity) or at least 75 min throughout the week (vigorous physical activity) or an equivalent combination of both were categorised as physically active^22^.

Variables under physical health status cover permanent disability or illness; physical, hearing, visual, and speech impairments; Activities of Daily Living (ADL); chronic diseases- hypertension, stroke, heart diseases, asthma, Chronic Obstructive Pulmonary Disease (COPD), cancer, diabetes and bones or joint problems; all categorised as yes and no. It also includes the presence of multi-morbidity coded as no morbidity, single morbidity and multi-morbidity defined as the presence of two or more morbidity condition, and Self Rated Health (SRH) recoded as poor for ‘very poor’ and ‘poor’, moderate for ‘fair’ and good for ‘good’ and ‘very good’.

Indicators of mental health status include state of cognitive health (poor and good); and presence of depression symptoms measured using Center for Epidemiologic Studies Depression (CES-D) Scale; diagnosed depression, trauma and mental problems, alzheimer & dementia, neurological psychiatric and mental impairment; all covariates in dichotomous form as yes and no. LASI used the cognition module of the Health and Retirement Study (HRS) to measure cognitive impairment across five domains- arithmetic function, memory, object naming of cognition orientation, and executive function. The lowest tenth percentile of the composite scores ranging from 0 to 43, denotes to ‘poor’ cognition.

Measures of social health and support include living arrangements (living alone, living with-spouse and/or others, spouse and children, children and others, and others only); having close family relationships, regularly meeting and talking with friend(s), participating in social activities, ill-treated or abused, ever received financial support and ever given financial support-all coded as yes and no; and facing everyday discrimination coded as no discrimination, one kind of discrimination and two or more kinds of discriminations. Individuals responding ‘none’ to the question “Among your family members/friends with whom would you say you have a close relationship with?” are referred to as ‘no’ to the close family relationship variable.

To understand the participation of individuals in social activities, a set of 11 questions about their experiences in their day-to-day life were asked in LASI– (1) Eat out of the house (Restaurant/Hotel); (2) Go to park/beach for relaxing/entertainment; (3) Play cards or indoor games; (4) Play outdoor games/sports/exercise/jog/yoga; (5) Visit relatives /friends; (6) Attend cultural performances/shows/Cinema; (7) Attend religious functions/events such as bhajan/satsang/prayer; (8) Attend political/community/organisation group meetings; (9) Read books/newspapers/magazines; (10) Watch television/listen radio; and (11) Use a computer for e-mail/net surfing. Each statement had seven response categories of ‘daily’, 'several times a week’, ‘once a week’, ‘several times a month’, ‘at least once a month’, or ‘rarely/once in a year’ and ‘never’. At least once a month or more participation in any of the 11 aforementioned activities is referred to as ‘yes’; otherwise ‘no’ for participating in social activities^22^.

To assess the everyday discrimination experienced by individuals, a set of 6 questions about their experiences in their day-to-day life were asked in LASI– (1) you are treated with less courtesy or respect than other people; (2) you are threatened or harassed; (3) people act as if they are afraid of you; (4) people act as if they think you are not smart; (5) you receive poorer service than other people at restaurants or stores; and (6) you receive poorer service or treatment than other people from doctors or hospitals. Each statement had six response categories ‘almost every day’, ‘at least once a week’, ‘a few times a month’, ‘a few times a year’, ‘less than once a year’, or ‘never’. The responses were then categorised as ‘no discrimination’, ‘one type of discrimination’, and ‘two or more types of discrimination’^22^.

### Statistical analysis

To systematically examine how self-rated life satisfaction varies by different background characteristics, health risk behaviours, and measures of physical, mental and social health, we conducted the descriptive statistical and cross-tabulation analysis. For examining the relationship, a chi-square test for the association at 5% level of significance was done. Further, we have applied hierarchical multiple logistic regression modelling to determine the adjusted effect of predictor variables on the likelihood of being satisfied from life, indicated by the outcome variable as self-rated life satisfaction. Throughout the regression analysis, the dichotomous outcomes variable coded “1” as satisfied and “0” as unsatisfied. Using the regression modelling, odds for satisfied life have been predicted across the socio-demographic, physical, mental and social health-related covariates.

The hierarchical regression Model-1 includes only the socio-demographic explanatory variables. Models 2 to 5, respectively, include health risk behaviours, measures of physical health, mental health and social well-being along with the socio-demographic predictors. All the variables categorised under five dimensions were taken into account in Model-6. All analysis was performed using STATA-15.

### Ethics approval and consent to participate

No ethical approval was required for this study. As this is the is a secondary based survey.

## Results

Table 1 presented the overall level of older persons’ perceived life satisfaction (LS), along with their varied socio-demographic features and health risk behaviours. Only on a small scale, it was determined that the level of high-LS decreased with ageing. According to the data, India's rural regions have (12.9%), i.e. a higher share of low-LS older people than its urban areas (8.2%). Other contributing factors such as education and wealth consumption expenditure) revealed a positive linear proportional relationship with life high-LS. Additionally, it was discovered that marriage had a big impact on self-reported LS. Currently, married individuals accounted for 50.8% high-LS among the older adult population; by contrast, widowed and divorced/separated individuals reported 40.6% and 34.3% of the older people, respectively. The status of LS among older persons in India varies significantly according to region-wise stratification. The western region of India reported the highest percentage of high-LS (61%), while the central region has the highest percentage of low-LS (14.7%). Across castes and tribes, SCs (16.2%) reported a higher prevalence of low-LS, followed by STs (12.2%) and OBCs (10.5%).

**Table 1:** Status of self-rated life satisfaction by socio-demographic characteristics and health risk behaviours among older adults in India.

Life satisfaction (LS)		Low LS	Medium LS	High LS	Sample (n)
Socio-demographic characteristics		(%)	(%)	(%)	
Age group***	45–59	9.6	40.9	49.5	33,958
	60–74	12.4	41.2	46.4	24,466
	75 and above	15.8	38.3	45.9	6435
Place of residence***	Rural	12.9	43.3	43.8	41,932
	Urban	8.2	35.1	56.8	22,927
Sex***	Male	10.8	40.6	48.6	34,702
	Female	11.9	40.8	47.3	30,157
Marital status***	Currently married	9.4	39.9	50.8	25,803
	Widowed	16.7	42.7	40.6	5981
	Divorced/separated/others	19.1	46.6	34.3	762
Religion***	Hindu	11.2	40.7	48.1	47,628
	Muslim	13.8	40.5	45.7	7699
	Christian	8.4	47.1	44.5	6429
	Others	9.3	37.0	53.8	3103
Caste/tribe***	Scheduled tribe	12.2	42.3	45.6	11,225
	Scheduled caste	16.2	43.0	40.9	10,835
	Other backward class	10.5	42.2	47.3	24,401
	None of the above	9.2	36.0	54.8	18,398
Education***	No schooling	14.8	45.0	40.2	30,348
	Less than 5 years complete	11.8	42.8	45.4	7405
	5–9 years complete	8.8	41.2	50.0	14,774
	10 or more years complete	4.6	26.9	68.5	12,332
Work status***	Currently working	11.0	42.8	46.2	32,188
	Worked in past but currently not working	13.4	39.8	46.8	14,927
	Never worked	10.2	37.5	52.3	17,744
Wealth quintile (MPCE)***	Poorest	14.3	44.0	41.7	12,764
	Poorer	12.1	42.6	45.4	13,031
	Middle	10.4	41.5	48.1	13,028
	Richer	9.4	39.7	50.9	13,091
	Richest	10.4	35.0	54.6	12,945
Region***	East	13.3	41.6	45.1	11,453
	North East	8.0	41.3	50.7	8401
	West	5.8	33.1	61.1	8821
	Central	14.7	38.9	46.4	8772
	North	9.7	34.5	55.8	11,891
	South	11.9	49.8	38.3	15,521
**Health risk behaviour**					
Smoked/smokeless tobacco***	No	10.2	39.6	50.2	40,723
	Yes	13.4	42.8	43.8	23,566
Ever drink alcohol***	No	11.2	40.2	48.6	52,701
	Yes	12.5	43.9	43.5	11,608
Physical activity***	Inactive	12.53	41.4	46.07	24,299
	Active	10.75	40.44	48.81	40,006
India		11.38	40.72	47.91	64,859

Significance level *p < 0.10, **p < 0.05, ***p < 0.01; *n* un-weighted sample.

Health risk behaviours were significantly associated (p < 0.001) with different levels of LS among older adults. Low-LS was more prevalent in tobacco smokers (13.4%) compared to those who have never consumed tobacco. Similarly, high-LS status is more prevalent among older adults who have never had alcohol (48.6%) and those who were physically active (48.8%).

Table 2 depicts the association of chronic diseases and physical health status (disabilities) with self-rated life satisfaction in older ages. Low-LS is most prevalent in respondents having speech impairment (29.3%), followed by those with physical impairment (24.8%), visual impairment (22.1%), ADL disabilities (21.4%), hearing disability (19.2%), and permanent disability or illness (16.8%). The level of life satisfaction varies with the presence of chronic diseases, with people with stroke (21%) showing the greatest share of people with low-LS, followed by people with COPD (17.4%), Asthma (16.4%), and Bronchitis (15.1%). The greatest share of people with high-LS was found in those with good self-rated health (63.9%). Whereas, the people who reported poor self-rated health showed the biggest share with low-LS (27.3%) followed by older adults with multi-morbid conditions (14.1%).

**Table 2:** Status of self-rated life satisfaction by physical health characteristics among older adults in India.

Life satisfaction (LS)		Low LS	Medium LS	High LS	Sample (n)
Physical health status		(%)	(%)	(%)	
Permanent disability or illness***	No	11.32	40.64	48.04	64,126
	Yes	16.76	47.89	35.34	733
Physical impairment***	No	10.74	40.59	48.67	62,025
	Yes	24.79	44.82	30.39	2545
Hearing impairment***	No	11.28	40.84	47.88	63,718
	Yes	19.21	36.61	44.18	852
Visual impairment***	No	11.08	40.71	48.21	62,837
	Yes	22.05	43.43	34.51	1733
Speech impairment***	No	11.33	40.8	47.87	64,365
	Yes	29.26	36.54	34.2	205
ADL limitation***	No	9.44	40.73	49.83	55,488
	Yes	21.39	41.07	37.54	9092
Chronic diseases					
Hypertension***	No	11.16	41.53	47.31	46,458
	Yes	12.02	38.81	49.17	18,225
Stroke***	No	11.22	40.76	48.02	63,607
	Yes	21.00	42.58	36.42	1082
Heart disease***	No	11.37	40.87	47.76	62,348
	Yes	11.94	38.9	49.16	2342
Asthma***	No	11.15	40.83	48.02	62,223
	Yes	16.38	40.08	43.53	2467
COPD***	No	11.31	40.85	47.84	63,995
	Yes	17.36	36.73	45.9	695
Bronchitis***	No	11.34	40.86	47.8	64,091
	Yes	15.14	35.7	49.16	599
Cancer***	No	11.37	40.83	47.8	64,259
	Yes	13.90	35.78	50.32	430
Diabetes***	No	11.59	41.17	47.24	56,386
	Yes	10.01	38.11	51.88	8294
Bones-joints problem***	No	10.74	40.3	48.96	55,396
	Yes	14.86	43.41	41.73	9295
Multi-morbidity***	No	10.33	40.9	48.76	35,755
	Single	11.78	41.93	46.29	17,865
	Multi (2 +)	14.13	38.72	47.15	11,056
Self-rated health (SRH)***	Poor	27.32	43.79	28.88	11,051
	Moderate	10.12	47.95	41.93	26,920
	Good	5.08	30.95	63.98	26,676

Significance level: *p < 0.10, **p < 0.05, ***p < 0.01; *n* un-weighted sample.

Table 3 presents significant associations between the level of self-rated life satisfaction and various mental health characteristics like major depression, cognitive health, and neuropsychiatry. Prevalence of low-LS is greater for older adults with poor cognitive health (19.7%), depression symptoms (21.9%), diagnosis of depression (34.9%), psychological trauma and mental problem (16.3%), Alzheimer's and dementia (36.8%), neurological psychiatric (22.7%), and mental impairment (27.6%) in comparison their counterparts.

**Table 3:** Status of self-rated life satisfaction by mental health characteristics among older adults in India.

Life satisfaction (LS)		Low LS	Medium LS	High LS	Sample (n)
Mental health status		(%)	(%)	(%)	
Cognitive health***	Good	9.88	40.33	49.78	51,666
	Poor	19.68	44.08	36.24	5757
Depression symptoms (CES-D scale)***	No	7.3	39.08	53.62	47,511
	Yes	21.89	45.29	32.81	16,280
Depression diagnosed***	No	11.25	40.78	47.98	64,339
	Yes	34.93	43.97	21.11	342
Psychological trauma and mental problem***	No	11.25	40.5	48.25	63,596
	Yes	16.27	49.65	34.07	1263
Alzheimer and dementia disease***	No	11.24	40.78	47.98	64,364
	Yes	36.85	44.18	18.97	317
Neurological psychiatric***	No	11.14	40.72	48.14	63,281
	Yes	22.66	44.12	33.21	1300
Mental impairment***	No	11.06	40.59	48.35	63,565
	Yes	27.6	50.47	21.93	1005

Significance level: *p < 0.10, **p < 0.05, ***p < 0.01; *n* un-weighted sample.

Table 4 shows the level of self-rated life satisfaction among older adults by their social health and support status. The respondents living alone showed the greatest shares of low-LS (30.4%), while those living with their spouse and children showed high-LS levels (52.49%). Those without any close family ties had a larger proportion with low-LS (15.77%) compared to those with family relations (10.97%). The respondents who do not regularly meet or talk to their friends also show a higher share of low-LS (12.3%) than their counterparts (9.3%). Data shows that those involved in social activities (50%) and provided financial support (54.3%) are more satisfied with their lives, reporting high-LS levels. Older adults receiving financial support are less satisfied (low-LS 14.3%) than those not financially dependent. Mistreated/ill-abused people reported greater low-LS. Older adults facing discrimination daily are more likely to be unsatisfied with their life.

**Table 4:** Status of self-rated life satisfaction by social health and support among older adults in India.

Life satisfaction (LS)		Low LS	Medium LS	High LS	Sample (n)
Social health & support		(%)	(%)	(%)	
Living arrangement***	Living alone	30.41	46.3	23.29	2288
	Living (spouse &others)	11.39	43.45	45.16	10,282
	Living (spouse & children)	8.75	38.75	52.49	37,309
	Living (children & others)	13.6	42.41	43.99	12,174
	Living (others only)	18.94	43.27	37.79	2806
Family close relation***	No	15.77	41.13	43.11	5128
	Yes	10.97	40.68	48.35	50,731
Friends meet or talk***	No	12.26	41.6	46.14	42,786
	Yes	9.32	39.21	51.47	21,341
Financial support received***	No	10.95	40.88	48.17	55,480
	Yes	14.27	41.13	44.61	8384
Financial support given***	No	11.5	41.34	47.16	58,794
	Yes	10.28	35.41	54.31	5065
Social activities***	No	18.49	43.81	37.7	10,310
	Yes	9.76	40.2	50.04	53,601
Ill-mistreated/abuse***	No	10.67	40.63	48.7	61,429
	Yes	26.09	44.94	28.97	2411
Everyday discrimination***	No	10.76	40.66	48.59	59,702
	One kind discrimination	15.26	46.74	38	2020
	Two or more kind of discriminations	24.46	40.6	34.94	2079

Significance level: *p < 0.10, **p < 0.05, ***p < 0.01; *n* un-weighted sample.

Table 5 and its supplementary table S1 presents estimates of the multiple logistic regression models that show how socio-demographic characteristics remain significant predictors of self-reported LS after adjusting for multiple variables. Model 1 shows that urban residents were 1.25 times (p < 0.001) more likely to have higher LS after adjusting for other socio-demographic characteristics. Older adults who are currently married were 1.76 times (p < 0.001) more likely to have a higher LS compared to widowed adults. Older adults who had completed ten or more years of schooling were more than twice as likely to have higher LS when compared to illiterates (OR: 2.42, p < 0.001). Older individuals who had never worked were 1.29 times (p < 0.001) more likely to have higher LS than those currently working. Older adults from ST, OBC and Others categories were more likely to be satisfied with their life.

**Table 5:** Multiple logistic regressions for factors influencing self-rated life satisfaction among older adults in India.

Covariates(®)		Model 1	Model 2	Model 3	Model 4	Model 5	Model 6
Socio-economic & demographic characteristics		AOR	AOR	AOR	AOR	AOR	AOR
Age group (45–59)	60–74	0.9***	0.92***	1.07**	0.94*	1.03	1.17***
	75 and above	0.84***	0.86***	1.28***	0.97	1.07	1.42***
Residence (rural)	Urban	1.25***	1.22***	1.19***	1.19***	1.12***	1.07*
Sex (female)	Male	0.99	1.19***	0.95	0.97	0.91**	1.03
Marital status (widow)	Currently married	1.76***	1.71***	1.7***	1.62***	1.56***	1.39**
	Divorced/separated/others	0.7***	0.68***	0.75***	0.7***	0.81***	0.86*
Religion (hindu)	Muslim	0.87***	0.86***	0.9**	0.84***	0.83***	0.86***
	Christian	1.63***	1.58***	1.61***	1.64***	1.56***	1.5***
	Others	1.22***	1.17**	1.22***	1.11	1.11	1.05
Caste (SC)	ST	1.61***	1.65***	1.42***	1.61***	1.62***	1.43***
	OBC	1.3***	1.27***	1.27***	1.27***	1.28***	1.21***
	None	1.27***	1.24***	1.24***	1.26***	1.23***	1.19***
Schooling level (no)	< 5 years complete	1.17***	1.17***	1.22***	1.09*	1.06	1.12**
	5–9 years complete	1.4***	1.38***	1.42***	1.3***	1.25***	1.27***
	10 or more years complete	2.42***	2.26***	2.12***	2.03***	1.95***	1.64***
Work status (working)	Worked in past but currently not	0.95	0.96	1.15***	1.02	0.96	1.17***
	Never worked	1.29***	1.28***	1.45***	1.36***	1.28***	1.4***
MPCE quintile (poorest)	Poorer	1.16***	1.17***	1.15***	1.14***	1.12***	1.12**
	Middle	1.24***	1.24***	1.22***	1.23***	1.24***	1.24***
	Richer	1.21***	1.22***	1.22***	1.16***	1.21***	1.25***
	Richest	1.14***	1.15***	1.17***	1.12**	1.22***	1.26***
Region (eastern)	North East	1.45***	1.51***	1.25***	1.28***	1.25***	1.03
	West	1.83***	1.81***	1.78***	1.81***	1.83***	1.56***
	Central	0.83***	0.82***	0.78***	0.87***	0.82***	0.77***
	North	1.25***	1.23***	1.15***	1.28***	1.18***	1.04
	South	1.04	1.01	1.12***	1.18***	1.06	1.08
**Health risk behaviours**							
Smoke and smokeless tobacco (yes)	No		1.31***				1.24***
Drink alcohol (yes)	No		1.18***				1.18***
Physical activity status (inactive)	Active		1.12***				1.01
**Physical health**							
Disability or illness (yes)	No			1.08			0.95
Physical impairment (yes)	No			1.37***			1.26***
Hearing impairment (yes)	No			1.03			0.95
Visual impairment (yes)	No			1.18**			0.95
Speech impairment (yes)	No			1.47**			1.38
ADL (yes)	No			1.49***			1.28***
Hypertension (yes)	No			0.9**			0.91**
Stroke (yes)	No			1.15			1.1
Heart disease (yes)	No			1.05			1.09
Asthma (yes)	No			1.09			1.1*
COPD(yes)	No			1.22*			1.15**
Cancer (yes)	No			1.26			1.25**
Diabetes (yes)	No			0.83***			0.88***
Bones-joints problem (yes)	No			1.05			0.99
SRH (poor)	Good			5.27***			4.54***
	Moderate			2.88***			2.61***
**Mental health**							
Cognitive health (poor)	Good				1.5***		1.27***
Depression symptoms (yes)	No				2.78***		2.23***
Depression diagnosed (yes)	No				1.59***		1.55**
Trauma & mental problem (yes)	No				1.42***		1.2**
Alzheimer & dementia (yes)	No				1.57***		1.57**
Neurological psychiatric (yes)	No				1.45***		1.05
Mental impairment (yes)	No				1.79***		1.17*
**Social health & support**							
Living arrangements (living alone)	Spouse and/or others					1.76***	1.8***
	Spouse& children					2.43***	2.5***
	Children& others					2.31***	2.32***
	Others only					1.84***	1.88***
Family relation (no)	Yes					1.06	1.13**
Friends meet/talk (no)	Yes					1.17***	1.14***
Social activities (no)	Yes					1.45***	1.27***
Mistreated or abused (yes)	No					2.77***	2.16***
Financial support received (no)	Yes					0.74***	0.81***
Financial support given (no)	Yes					1.34***	1.25***
Discrimination (no)	One kind of					0.66***	0.79***
	Two or more kind of					0.66***	0.85**
Constant		2.86	1.91	0.176	0.131	0.449	0.009
Significant		p < 0.01	p < 0.01	p < 0.01	p < 0.01	p < 0.01	p < 0.01
Psudo-R^2^		0.053	0.06	0.11	0.092	0.078	0.154

Significance level: *p < 0.10, **p < 0.05, ***p < 0.01; Model-1: Socio-economic and demographic characteristics, Model-2: Health risk behaviours, Model-3: Physical health status, Model-4: Mental health status, Model-5: Social health and support and model-6: All factors included. All models are adjusted for socio-demographic characteristics.

® Reference category with odds ratio one.

Model 2 depicts the odds of satisfied life when controlling for various health risk behaviours and other socio-demographic factors. The older respondents who had never consumed tobacco were 1.31 times (p < 0.001) more likely to have higher LS when compared to respondents who consume tobacco. Similarly as per modal 2, older adults who have never had alcohol and those who are physically active were 1.18 (p < 0.001) and 1.12 times (p < 0.001) more likely to report higher LS than their counterparts, respectively.

Model 3 indicates the odds of having high LS among the older population when controlled for various physical disabilities and diseases along with socio-demographic factors. The results show that a state of good physical health is significantly associated with higher reporting of LS. The level of LS for adults without any physical, visual or speech impairment was likely to be higher by 1.37 times (p < 0.001), 1.18 times (p < 0.01), and 1.47 times (p < 0.01), respectively, when compared to those with these impairments. In the case of activities of daily living (ADL), the respondents who can independently do their daily activities are almost 1.5 times (p < 0.001) times more likely to have higher LS than the older adults with ADL disabilities. Most importantly, self-rated health (SRH) was found to be a significant factor in determining LS, with those reporting good SRH being five times more likely to have a satisfied life (OR: 5.27, p < 0.001) when compared to older adults with poor SRH.

Model 4 indicates the odds of reporting higher LS after controlling for several mental health variables in addition to the background characteristics. The adjusted odds for higher LS in the case of good cognitive health is 1.5 times (OR: 1.5, p < 0.001) that of someone with a poor one. Older adults without any depressive symptoms on the CES-D scale are almost thrice more likely (OR: 2.78, p < 0.001) to have higher LS than those who demonstrate symptoms of depression. Similarly, respondents who reported no diagnosis of depression or Alzheimer's are almost 1.6 times more likely (OR: 1.59 & 1.57; p < 0.001 & p < 0.001; respectively) to report satisfied life. People who were not suffering from trauma or any neuro-psychiatric problems were at least 1.4 times more likely (OR: 1.42 & 1.45, p < 0.001 & p < 0.001, respectively) to report higher LS.

Model 5 represents the odds of reporting higher LS for older adults after controlling for social health and support variables on top of the various background factors. The model revealed that living arrangements, meeting or talking with friends, being involved in social activities, giving or receiving financial support, and suffering from abuse or discrimination are significantly associated with LS in a social context. Older adults living with spouses and children are 2.4 times more likely (OR: 2.43, p < 0.001) to have higher LS than those living alone. The odds of being satisfied from life is 2.8 (p < 0.001) times for the people who were not mistreated or abused in the last year with respect to their counterparts. Older adults regularly participating in social activities or providing financial support were more likely (OR: 1.45, p < 0.001 and OR: 1.34, p < 0.001, respectively) to be satisfied with their life. Older individuals receiving financial support or facing discrimination in daily life were more likely to be unsatisfied with their life (OR: 0.74, p < 0.001 and OR: 0.66, p < 0.001, respectively).

Model 6 includes all of the explanatory variables considered in our study and gives adjusted odds of higher LS overall. Firstly, LS among older adults increases with age when all adjusting factors were included in the model. Life satisfaction for respondents in the age group 60–74 and 75 above are more likely by odds of 1.17 (p < 0.001) and 1.42 (p < 0.001) times, respectively, when compared to adults between 45 and 59 years of age. People who have never used tobacco and non-alcoholics are 1.24 (p < 0.001) and 1.18 (p < 0.001) times, respectively, more likely to report higher satisfaction in life. The respondents without ADL disabilities are 1.28 times more likely to have higher LS than those suffering from ADL limitations. People who reported good SRH are 4.5 times more likely (OR: 4.45, p < 0.001) to report high LS among older adults than those with poor SRH. Similarly, in the case of mental health domains, respondents with poor cognitive health, depression symptoms, and other mental problems show a negative association with LS. In the context of social health and support, the respondents who showed positive social health are more likely to be satisfied among older adults with their life.

Based on all six regression models shown in Table 5, the contributing determinants of LS among Indian elders can be decomposed accordingly. The results unveiled that the socio-demographic (model 1) only made up 5.3% of the total contribution to higher LS. When combined with health risk behaviours (model 2), it rose up to 6% in explaining LS among older adults. Model 3 contributed 11% when physical health determinants were combined with background factors. Furthermore, model 4 and model 5, based on mental health, social health & support along with background characteristics, contributed 9.2% and 7.8% to LS, respectively. Finally, all the overall covariates model with socio-demographic, health risk behaviour, physical, social, and mental health (model 6) contributed to 15.4% in determining overall LS in older adults of India.

## Discussion

Life satisfaction is affected by socio-demographic status, physical and mental health, social activity partition, living arrangements and major life events. There are several affirmations on the relationship between the socio-demographic variable and health risk behaviour with life satisfaction. Several studies found a *U*-shape relationship between the aged 16–65 and their life satisfaction^23,24^; life satisfaction decline rapidly with ageing. Our finding confirms that with increasing age, life satisfaction declines^25,26^ at the crude level. But it contradicts when adjusting with various socio-demographical and health-related factors as affected in real life, it revealed life satisfaction increasing with age. The gender difference did not show significant differences in life satisfaction, which oppose several studies^27,28^. The wealth quintile is the strongest predictor for life satisfaction, consistent with studies that show greater well-being in a rich country than in a poor country^28^. Our study also examines that when the year of schooling increase, older people live better quality lives and are satisfied with them. We observed that positive health behaviours (no use of tobacco or alcohol and physical activities) positively influence life satisfaction. In contrast, some studies did not find a relation between health risk behaviours and life satisfaction^29^.

Furthermore, the study has shown the significant effect that chronic diseases and physical disabilities or functional limitations could have on life satisfaction. Since, with increasing age, health conditions deteriorate and deplete the functionality of organs, hence it affects elders not only physically but also mentally, causing dissatisfaction with their lives^30,31^. There has been evidence that the more the number of medical conditions, the lesser the life satisfaction among older adult^32,33^. The degree of happiness increases with an increase in functional abilities to do activities of daily living and a decrease in chronic diseases^34^. Low life satisfaction is associated with low ADL and IADL and affects the overall quality of life^5,35^. Most people surviving a stroke suffer from post-stroke depression which leads to a decrease in life satisfaction^36^. Older people with a history of pulmonary diseases, including asthma, bronchitis or COPD, have a substantial impact on their life satisfaction^37,38^, which in a way, is related to high neuroticism personality traits^39^. Also, one’s self-assessment of their health conditions at an older age has emerged as a major determinant of life satisfaction^40,41^.

This study has shown that mental health problems are significantly associated with reductions in life satisfaction and presented evidence of a reciprocal relationship between poor mental health status and life satisfaction. There is a clear difference in life satisfaction between individuals with poor mental health and those without symptoms. While most existing studies on this topic were performed on young individuals, we have examined the relationship between poor mental health conditions and life satisfaction in later life and older ages^42–44^. Many preceding studies have shown the presence of depression with low life satisfaction; our study tried to take a step further by pointing to the undiagnosed nature of depression as well^45–47^. While substantial research has been conducted on life satisfaction and mental health, research has focused on the influence of life satisfaction on mental health in nearly all studies. Bar a few exceptions, our study is a niche in the manner it suggests that poor mental health and reduced life satisfaction mutually influence each other^48,49^. The findings of this study are consistent with the conclusions that exposure to psychological trauma among older adults is associated with lower levels of life satisfaction^50^. People suffering from Alzheimer's and other neuropsychiatric diseases showed extremely low levels of life satisfaction, which conflicts with findings from a few clinical studies and exerted the importance of self-ratings in older patients^51–53^.

The study has focused on examining the association between various aspects of social dynamics and self-rated life satisfaction in older Indian adults. Our first point in question was the relationship between living arrangements and life satisfaction, and the findings of our study are consistent with previous studies^17,54,55^. As older adults behold new vulnerabilities with age and a further decline in functioning, the types of living arrangements act as a powerful tool to define social roles and provide support functions^56,57^. This study showed that older adults who live alone or with people other than a spouse or children have lower life satisfaction than those who share a roof with a partner or children. This is consistent with the results of other studies^58–60^. Secondly, the study affirmed previous findings, which indicated the importance of family relations and support network to life satisfaction for older people^61,62^. The bonding with family bond was associated with higher life satisfaction. Due to a familial sense of togetherness, family ties contribute to higher emotional well-being and, subsequently, greater satisfaction from one’s life^63,64^. Individuals are embedded into relationships with their friends, constituting their social capital. These relations are great resources of well-being and support and can significantly impact the perception of one’s life^65^. A possible touted reason from previous studies is that friends provide companionship, intimacy, and help, increasing an individual's life satisfaction^66^.

Financial well-being among older adults is fundamental to their participation in social activities essential for higher life satisfaction. Social activities gather social capital, which encompasses different aspects of family and social relationships, and, therefore, could explain life satisfaction among older adults, at least on a rudimentary level^67^. Some adults who otherwise would be satisfied with their lives might end up worried and uncomfortable due to financial dependence^68,69^. The study also assessed social engagement as a determinant of life satisfaction among older individuals. There is a substantial difference when it comes to the role of social engagement for functional and dysfunctional adults. Older individuals with frailties and disabilities can enjoy successful ageing by positively maintaining high levels of active social engagement^70,71^. People who have been victims of any form of abusive behaviour are more likely to perceive dissatisfaction in life due to a lack of emotional support^72^. Previous studies have conferred the life satisfaction approach in estimating the costs of domestic violence as it can closely address the post-trauma state of a survivor^73^. Another very important reason to consider abuse as a determinant is that although violence negatively affects the life satisfaction of both women and men, women are more likely to experience violence and discrimination than men during their life course^74,75^.

The study also analysed the association between a multitude of discriminations and the life satisfaction of older Indians. The results are consistent in portraying the negative impact discrimination of any form can have on the life satisfaction of individuals as well as on their community. One possible explanation could be that the experience of discrimination provokes stress, and stress can effectively lower overall life satisfaction^76,77^. Therefore, the results of our study can also cement the viewpoint of Urie Bronfenbrenner's Ecological Systems theory, which has been one of the most widely accepted explanations of the influence social environments can have on individuals' behaviour and their lateral development^78,79^. Finally, considering the overall picture of the study, life satisfaction is a subjective phenomenon that depends on numerous factors, from unwanted things to wanted achievements. It is a well-understood indicator of health and mortality. Accordingly to WHO, four factors directly influencing LS levels among older adults are mental health, physical health condition, social relationships, and environment^19^. Our study has provided further evidence to explain the LS level by socio-demographic background, health risk behaviour, physical, mental and social health & support. The descriptive attributes found substantial determinates for the LS among older adults. These findings are also consistent with a few earlier studies^5,80–82^. This study's findings are consistent with strong epidemiological and physiological pieces of evidence in the literature. In our study, with the help of regression analysis, models show that socio-demographic and health risk behaviour factors influence self-reported LS. However, the utmost influential covariates were physical, mental, and social health & support. Respondents who were less educated, from the poorest background, and consumed tobacco or alcohol were found to be more likely to have lower LS, which was quite similar to the findings from many of the previous studies^83,84^. This study also likewise discussed the main influencing determinants for a better LS level. Physical impairment, ADL limitations, chronic diseases, SRH, cognitive health, depression, neuropsychiatric problems, living arrangement, social activities, mistreatment, discrimination, and financial support all play important roles in the construction of one's life satisfaction. Earlier studies have also provided similar influencing determinates for LS in India and outside of India^5,13,82,85–87^. Ultimately, among the various determinates for life satisfaction, physical conditions, mental health and social dynamics are important components to achieving ideal or complete life satisfaction among older adults in India. However, despite the elders’ relatively poor physical and mental health conditions with ageing, our study shows that most older Indian adults are satisfied with their lives.

### Limitations

The result of this study must be considered in light of several limitations. Firstly, this study has taken self-reported life satisfaction that can be misreported because of social stigma. Secondly, The NCD prevalence and disabilities may be under-reported as it is based on the individuals’ self-reporting as chronic and social stigma. Lastly, for the health risk behavioural factors such as smoking and drinking, we have used the ever-smoked and ever-drink, which might not be that strong indicator without information on the intensity and duration of smoking or drinking. When it comes to quantitative methods, it is not easy to draw causal relationships between life satisfaction and other variables. However, this study can become an initiation for extensive qualitative investigation, the results of which already affirm the truth and consistency of certain outcomes.

## Supplementary Information

Supplementary Table S1.

## Data Availability

The study uses data from LASI Wave-1 data collected by the nodal agency International Institute for Population Sciences (IIPS), Mumbai, on behalf of the Government of India. Data were de-identified which is publicly available to the researchers and policymakers upon formal request to the nodal agency IIPS. To access the data request (link to the data request document LASI_DataRequestForm_0.pdf (iipsindia.ac.in)) and for information related to the LASI data set Longitudinal Ageing Study in India (LASI) | International Institute for Population Sciences (IIPS) (iipsindia.ac.in).

## Abbreviations

LS: Life satisfaction
LASI: Longitudinal Ageing Survey in India
AOR: Adjusted odds ratio
ADL: Activities of daily living
SRH: Self-rated health
COPD: Chronic obstructive pulmonary disease
MPCE: Monthly per capita expenditure
