# Supplementary material for: An assessment of self-rated life satisfaction and its correlates with physical, mental and social health status among older adults in India
Source: Sci Rep. 2023 Jun 5;13:9117. doi: 10.1038/s41598-023-36041-3 (PMC10241830; doi:10.1038/s41598-023-36041-3)
Supplement: Supplementary file 1 — Supplementary Table S1. [file 41598_2023_36041_MOESM1_ESM.docx]

| **Table: S1 : Multiple logistic regreesion for factors influencing Self Rated Life Satisfaction (SRLS) among older adults in India.** | | | | | | | | | | | | | | | | | | | |  |
| --- | --- | --- | --- | --- | --- | --- | --- | --- | --- | --- | --- | --- | --- | --- | --- | --- | --- | --- | --- | --- |
|  |  | **Model 1** | | | **Model 2** | | | **Model 3** | | | **Model 4** | | | **Model 5** | | | **Model 6** | | |  |
| **Socio-Demographic(® )** |  | **AOR** | **95% CI** | | **AOR** | **95% CI** | | **AOR** | **95% CI** | | **AOR** | **95% CI** | | **AOR** | **95% CI** | | **AOR** | **95% CI** | |  |
| Age group (45-59) | 60-74 | 0.90 | 0.85 | 0.96 | 0.92 | 0.86 | 0.97 | 1.07 | 1.01 | 1.14 | 0.94 | 0.88 | 1.00 | 1.00 | 0.94 | 1.07 | 1.17 | 1.08 | 1.25 | |
|  | 75 and above | 0.84 | 0.77 | 0.92 | 0.86 | 0.78 | 0.95 | 1.28 | 1.15 | 1.41 | 0.97 | 0.87 | 1.08 | 0.98 | 0.89 | 1.08 | 1.42 | 1.26 | 1.60 | |
| Residence (Rural) | Urban | 1.25 | 1.17 | 1.33 | 1.22 | 1.14 | 1.30 | 1.19 | 1.11 | 1.27 | 1.19 | 1.11 | 1.28 | 1.14 | 1.06 | 1.22 | 1.07 | 1.00 | 1.16 | |
| sex (Female) | Male | 0.99 | 0.93 | 1.06 | 1.19 | 1.10 | 1.28 | 0.95 | 0.89 | 1.02 | 0.97 | 0.90 | 1.05 | 0.94 | 0.87 | 1.00 | 1.03 | 0.94 | 1.13 | |
| Marital status (Widow) | Currently married | 1.76 | 1.64 | 1.87 | 1.71 | 1.61 | 1.83 | 1.70 | 1.59 | 1.82 | 1.62 | 1.51 | 1.75 | 1.52 | 1.19 | 1.95 | 1.39 | 1.05 | 1.84 | |
|  | Divorced/Separated/Deserted/ Others | 0.70 | 0.62 | 0.80 | 0.68 | 0.60 | 0.78 | 0.75 | 0.66 | 0.86 | 0.70 | 0.61 | 0.82 | 0.81 | 0.70 | 0.94 | 0.86 | 0.73 | 1.01 | |
| Religion (Hindu) | Muslim | 0.87 | 0.80 | 0.94 | 0.86 | 0.79 | 0.93 | 0.90 | 0.83 | 0.98 | 0.84 | 0.77 | 0.92 | 0.85 | 0.78 | 0.93 | 0.86 | 0.78 | 0.94 | |
|  | Christian | 1.63 | 1.43 | 1.87 | 1.58 | 1.38 | 1.81 | 1.61 | 1.40 | 1.84 | 1.64 | 1.42 | 1.90 | 1.58 | 1.38 | 1.81 | 1.50 | 1.29 | 1.74 | |
|  | Others | 1.22 | 1.05 | 1.40 | 1.17 | 1.01 | 1.35 | 1.22 | 1.05 | 1.42 | 1.11 | 0.95 | 1.30 | 1.15 | 0.99 | 1.33 | 1.05 | 0.89 | 1.24 | |
| Caste (SC) | ST | 1.61 | 1.46 | 1.78 | 1.65 | 1.49 | 1.82 | 1.42 | 1.28 | 1.57 | 1.61 | 1.44 | 1.80 | 1.55 | 1.40 | 1.72 | 1.43 | 1.28 | 1.61 | |
|  | OBC | 1.30 | 1.21 | 1.40 | 1.27 | 1.19 | 1.37 | 1.27 | 1.18 | 1.37 | 1.27 | 1.18 | 1.38 | 1.29 | 1.20 | 1.39 | 1.21 | 1.11 | 1.32 | |
|  | None | 1.27 | 1.17 | 1.38 | 1.24 | 1.14 | 1.35 | 1.24 | 1.14 | 1.35 | 1.26 | 1.15 | 1.39 | 1.23 | 1.13 | 1.34 | 1.19 | 1.08 | 1.31 | |
| Schooling level (NO) | <5 years complete | 1.17 | 1.07 | 1.28 | 1.17 | 1.07 | 1.28 | 1.22 | 1.11 | 1.34 | 1.09 | 0.98 | 1.20 | 1.11 | 1.02 | 1.22 | 1.12 | 1.01 | 1.24 | |
|  | 5-9 years complete | 1.40 | 1.30 | 1.51 | 1.38 | 1.28 | 1.49 | 1.42 | 1.31 | 1.53 | 1.30 | 1.19 | 1.42 | 1.31 | 1.21 | 1.42 | 1.27 | 1.16 | 1.39 | |
|  | 10 or more years complete | 2.42 | 2.18 | 2.68 | 2.26 | 2.03 | 2.51 | 2.12 | 1.90 | 2.36 | 2.03 | 1.81 | 2.28 | 2.12 | 1.90 | 2.36 | 1.64 | 1.46 | 1.85 | |
| Work Status (Working) | Worked in past but currently not | 0.95 | 0.88 | 1.02 | 0.96 | 0.89 | 1.03 | 1.15 | 1.07 | 1.23 | 1.02 | 0.94 | 1.10 | 0.95 | 0.89 | 1.02 | 1.17 | 1.08 | 1.27 | |
|  | Never Worked | 1.29 | 1.20 | 1.40 | 1.28 | 1.19 | 1.39 | 1.45 | 1.34 | 1.57 | 1.36 | 1.25 | 1.48 | 1.26 | 1.16 | 1.36 | 1.40 | 1.28 | 1.54 | |
| MPCE Quintile (Poorest) | Poorer | 1.16 | 1.07 | 1.26 | 1.17 | 1.08 | 1.26 | 1.15 | 1.06 | 1.25 | 1.14 | 1.05 | 1.25 | 1.14 | 1.06 | 1.24 | 1.12 | 1.03 | 1.23 | |
|  | Middle | 1.24 | 1.14 | 1.34 | 1.24 | 1.14 | 1.35 | 1.22 | 1.12 | 1.33 | 1.23 | 1.12 | 1.35 | 1.25 | 1.15 | 1.36 | 1.24 | 1.12 | 1.36 | |
|  | Richer | 1.21 | 1.11 | 1.31 | 1.22 | 1.12 | 1.32 | 1.22 | 1.12 | 1.34 | 1.16 | 1.06 | 1.28 | 1.24 | 1.14 | 1.35 | 1.25 | 1.13 | 1.38 | |
|  | Richest | 1.14 | 1.05 | 1.25 | 1.15 | 1.05 | 1.26 | 1.17 | 1.07 | 1.29 | 1.12 | 1.01 | 1.23 | 1.26 | 1.15 | 1.38 | 1.26 | 1.14 | 1.40 | |
| Region (Eastern) | North East | 1.45 | 1.29 | 1.63 | 1.51 | 1.34 | 1.70 | 1.25 | 1.10 | 1.41 | 1.28 | 1.13 | 1.45 | 1.21 | 1.08 | 1.37 | 1.03 | 0.90 | 1.17 | |
|  | West | 1.83 | 1.64 | 2.04 | 1.81 | 1.63 | 2.02 | 1.78 | 1.59 | 1.99 | 1.81 | 1.61 | 2.04 | 1.67 | 1.50 | 1.87 | 1.56 | 1.38 | 1.77 | |
|  | Central | 0.83 | 0.76 | 0.90 | 0.82 | 0.75 | 0.89 | 0.78 | 0.71 | 0.85 | 0.87 | 0.79 | 0.96 | 0.83 | 0.76 | 0.91 | 0.77 | 0.69 | 0.85 | |
|  | North | 1.25 | 1.15 | 1.37 | 1.23 | 1.13 | 1.35 | 1.15 | 1.05 | 1.27 | 1.28 | 1.16 | 1.41 | 1.11 | 1.01 | 1.22 | 1.04 | 0.94 | 1.16 | |
|  | South | 1.04 | 0.96 | 1.13 | 1.01 | 0.93 | 1.10 | 1.12 | 1.03 | 1.22 | 1.18 | 1.08 | 1.29 | 0.98 | 0.90 | 1.07 | 1.08 | 0.98 | 1.19 | |
| **Health Risk Behaviour** |  |  |  |  |  |  |  |  |  |  |  |  |  |  |  |  |  |  |  | |
| smoke and smokless tobacco(Yes) | No |  |  |  | 1.31 | 1.23 | 1.39 |  |  |  |  |  |  |  |  |  | 1.24 | 1.16 | 1.34 | |
| Drink alcohol (Yes) | No |  |  |  | 1.18 | 1.09 | 1.28 |  |  |  |  |  |  |  |  |  | 1.18 | 1.08 | 1.30 | |
| Physically Active status (Inactive) | Active |  |  |  | 1.12 | 1.06 | 1.19 |  |  |  |  |  |  |  |  |  | 0.95 | 0.89 | 1.02 | |
| **Physical Health** |  |  |  |  |  |  |  |  |  |  |  |  |  |  |  |  |  |  |  | |
| Per phys_disability_illness(Yes) | No |  |  |  |  |  |  | 1.08 | 0.86 | 1.35 |  |  |  |  |  |  | 0.95 | 0.73 | 1.23 | |
| Physical Impairment (Yes) | No |  |  |  |  |  |  | 1.37 | 1.22 | 1.55 |  |  |  |  |  |  | 1.26 | 1.10 | 1.45 | |
| Hearing Impairment (Yes) | No |  |  |  |  |  |  | 1.03 | 0.83 | 1.26 |  |  |  |  |  |  | 0.95 | 0.74 | 1.22 | |
| Visual Impairment (Yes) | No |  |  |  |  |  |  | 1.18 | 1.01 | 1.37 |  |  |  |  |  |  | 0.95 | 0.80 | 1.14 | |
| Speech Impairment (Yes) | No |  |  |  |  |  |  | 1.47 | 1.04 | 2.07 |  |  |  |  |  |  | 1.38 | 0.90 | 2.10 | |
| Activities of daily living (Yes) | No |  |  |  |  |  |  | 1.49 | 1.39 | 1.60 |  |  |  |  |  |  | 1.28 | 1.18 | 1.39 | |
| Hypertention(Yes) | No |  |  |  |  |  |  | 0.90 | 0.85 | 0.96 |  |  |  |  |  |  | 0.91 | 0.84 | 0.98 | |
| Stoke(Yes) | No |  |  |  |  |  |  | 1.15 | 0.97 | 1.38 |  |  |  |  |  |  | 1.10 | 0.90 | 1.35 | |
| Heart disease(Yes) | No |  |  |  |  |  |  | 1.05 | 0.92 | 1.20 |  |  |  |  |  |  | 1.09 | 0.94 | 1.27 | |
| Asthma(Yes) | No |  |  |  |  |  |  | 1.09 | 0.96 | 1.23 |  |  |  |  |  |  | 1.10 | 0.96 | 1.26 | |
| COPD(Yes) | No |  |  |  |  |  |  | 1.22 | 0.98 | 1.52 |  |  |  |  |  |  | 1.15 | 0.90 | 1.48 | |
| Cancer(Yes) | No |  |  |  |  |  |  | 1.26 | 0.95 | 1.67 |  |  |  |  |  |  | 1.25 | 0.92 | 1.71 | |
| Diabeates(Yes) | No |  |  |  |  |  |  | 0.83 | 0.76 | 0.91 |  |  |  |  |  |  | 0.88 | 0.79 | 0.97 | |
| Bones-Joints Problem(Yes) | No |  |  |  |  |  |  | 1.05 | 0.97 | 1.13 |  |  |  |  |  |  | 0.99 | 0.91 | 1.08 | |
| SRH (Poor) | Better |  |  |  |  |  |  | 5.27 | 4.86 | 5.70 |  |  |  |  |  |  | 4.54 | 4.15 | 4.97 | |
|  | Normal |  |  |  |  |  |  | 2.88 | 2.70 | 3.07 |  |  |  |  |  |  | 2.61 | 2.42 | 2.81 | |
| **Mental Health** |  |  |  |  |  |  |  |  |  |  |  |  |  |  |  |  |  |  |  | |
| Cognitive health (Poor) | Good |  |  |  |  |  |  |  |  |  | 1.50 | 1.37 | 1.63 |  |  |  | 1.27 | 1.16 | 1.39 | |
| Depression Symptoms (Yes) | No |  |  |  |  |  |  |  |  |  | 2.78 | 2.62 | 2.95 |  |  |  | 2.23 | 2.09 | 2.37 | |
| Depression Diagnosed (Yes) | No |  |  |  |  |  |  |  |  |  | 1.59 | 1.13 | 2.23 |  |  |  | 1.55 | 1.09 | 2.20 | |
| Trauma & mental problem (Yes) | No |  |  |  |  |  |  |  |  |  | 1.42 | 1.19 | 1.68 |  |  |  | 1.20 | 1.00 | 1.44 | |
| Alzhemer & Dementia (Yes) | No |  |  |  |  |  |  |  |  |  | 1.57 | 1.13 | 2.19 |  |  |  | 1.57 | 1.11 | 2.22 | |
| Neurological Psychiatric(Yes) | No |  |  |  |  |  |  |  |  |  | 1.45 | 1.18 | 1.79 |  |  |  | 1.05 | 0.85 | 1.31 | |
| Mental Impairment(Yes) | No |  |  |  |  |  |  |  |  |  | 1.79 | 1.49 | 2.16 |  |  |  | 1.17 | 0.95 | 1.44 | |
| **Social Health & Support** |  |  |  |  |  |  |  |  |  |  |  |  |  |  |  |  |  |  |  | |
| Living Aarrangements (Living alone) | living (spouse and/or others) |  |  |  |  |  |  |  |  |  |  |  |  | 1.76 | 1.35 | 2.29 | 1.80 | 1.33 | 2.42 | |
|  | living ( spouse and children) |  |  |  |  |  |  |  |  |  |  |  |  | 2.43 | 1.87 | 3.15 | 2.50 | 1.86 | 3.35 | |
|  | living ( children and others) |  |  |  |  |  |  |  |  |  |  |  |  | 2.31 | 2.05 | 2.60 | 2.32 | 2.03 | 2.66 | |
|  | living ( others only) |  |  |  |  |  |  |  |  |  |  |  |  | 1.84 | 1.58 | 2.14 | 1.88 | 1.58 | 2.25 | |
| Family Relation (No) | Yes |  |  |  |  |  |  |  |  |  |  |  |  | 1.06 | 0.97 | 1.16 | 1.13 | 1.01 | 1.25 | |
| Friends meet or talk (No) | Yes |  |  |  |  |  |  |  |  |  |  |  |  | 1.17 | 1.09 | 1.25 | 1.14 | 1.06 | 1.23 | |
| Social Activites (No) | Yes |  |  |  |  |  |  |  |  |  |  |  |  | 1.45 | 1.35 | 1.55 | 1.27 | 1.17 | 1.37 | |
| Ill-mistreated or Abuse (Yes) | No |  |  |  |  |  |  |  |  |  |  |  |  | 2.77 | 2.50 | 3.07 | 2.16 | 1.92 | 2.43 | |
| Financial support received (No) | Yes |  |  |  |  |  |  |  |  |  |  |  |  | 0.74 | 0.69 | 0.80 | 0.81 | 0.74 | 0.88 | |
| Financial Support given (No) | Yes |  |  |  |  |  |  |  |  |  |  |  |  | 1.34 | 1.19 | 1.51 | 1.25 | 1.09 | 1.42 | |
| Everyday Discrimination (No) | One kind |  |  |  |  |  |  |  |  |  |  |  |  | 0.66 | 0.58 | 0.75 | 0.79 | 0.68 | 0.92 | |
|  | 2 or more kind |  |  |  |  |  |  |  |  |  |  |  |  | 0.66 | 0.58 | 0.74 | 0.85 | 0.73 | 0.99 | |
| **Constant** |  | **2.86** | | | **1.91** | | | **0.176** | | | **0.131** | | | **0.449** | | | **0.009** | | |  |
| **Degrees of freedom** |  | **26** | | | **29** | | | **42** | | | **33** | | | **38** | | | **64** | | |  |
| **Significant** |  | **p<0.01** | | | **p<0.01** | | | **p<0.01** | | | **p<0.01** | | | **p<0.01** | | | **p<0.01** | | |  |
| **R^2** |  | **0.053** | | | **0.06** | | | **0.11** | | | **0.092** | | | **0.078** | | | **0.154** | | |  |

*Model-1: Socio-economic and demographic characteristics, Model-2: Health risk behaviours, Model-3: Physical health status, Model-4: Mental health status, Model-5: Social health and support and model-6: All factors included. All models are adjusted for socio-demographic characteristics.*

*®: Reference category with odds ratio one.*
